# Detection of crown-like structures in breast adipose tissue and clinical outcomes among African-American and White women with breast cancer

**DOI:** 10.1186/s13058-020-01308-4

**Published:** 2020-06-17

**Authors:** Maret L. Maliniak, Aswathy Miriam Cheriyan, Mark E. Sherman, Yuan Liu, Keerthi Gogineni, Jiaqi Liu, Jiabei He, Uma Krishnamurti, Jasmine Miller-Kleinhenz, Ryan Ashiqueali, Jinjing He, Rami Yacoub, Lauren E. McCullough

**Affiliations:** 1 Department of Epidemiology, Rollins School of Public Health, Emory University, Atlanta, GA USA; 2 Allegheny Health Network, Pittsburgh, PA USA; 3 Department of Health Sciences Research, Mayo Clinic, Jacksonville, FL USA; 4 Department of Biostatistics and Bioinformatics, Rollins School of Public Health, Emory University, Atlanta, GA USA; 5 Winship Cancer Institute of Emory University, Atlanta, GA USA; 6 Department of Hematology and Medical Oncology, Emory University School of Medicine, Atlanta, GA USA; 7 Department of Pathology and Laboratory Medicine, Emory University School of Medicine, Atlanta, GA USA

**Keywords:** Crown-like structures, Breast adipose tissue, Obesity, Progression-free survival, Breast cancer outcome disparity

## Abstract

**Background:**

Crown-like structures in breast adipose tissue (CLS-B), composed of necrotic adipocytes encircled by macrophages, are associated with obesity and hypothesized to worsen breast cancer prognosis; however, data are sparse, particularly in multi-racial populations.

**Methods:**

We assessed specimens for CLS-B from 174 African-American and 168 White women with stage I–III breast cancer treated by mastectomy. Benign breast tissue from an uninvolved quadrant was immunohistochemically stained for CD68 to determine CLS-B presence and density (per cm^2^ of adipose tissue). Demographic and lifestyle factors, collected via medical record review, were analyzed for associations with CLS-B using logistic regression. Multivariable Cox proportional hazards models were used to compute hazard ratios (HRs) and 95% confidence intervals (CIs) for associations between CLS-B and overall (OS) or progression-free (PFS) survival.

**Results:**

Detection of any CLS-B was similar between African-American (32%) and White (29%) patients with no evidence of an association between race and CLS-B in multivariable models (OR = 0.82, 95% CI = 0.49–1.36). Detection of CLS-B was associated with obesity (OR = 4.73, 95% CI = 2.48–9.01) and age ≥ 60 years at diagnosis (OR = 1.78, 95% CI = 0.99–3.21). There was some evidence of associations with parity and current smoking status. Detection of CLS-B was not associated with OS (HR = 1.02, 95% CI = 0.55–1.87) or PFS (HR = 0.99, 95% CI = 0.59–1.67).

**Conclusions:**

Our results show a strong, positive association between BMI and CLS-B in non-tumor tissue similar to previous findings. Detection of CLS-B did not vary by race and was not associated with worse OS or PFS.

## Background

Obesity is an established risk factor for postmenopausal breast cancer, notably hormone receptor-positive cancers [1, 2], and has been linked to a higher risk of recurrence and mortality regardless of menopausal and hormone receptor status [1, 3, 4]. Both systemic and local effects of obesity interact and contribute to the development and progression of breast cancer although mechanisms are not fully understood [1, 5]. Among postmenopausal women, adipose tissue is the main source of circulating estrogens, accounting for the link between obesity and increased breast cancer risk [6]. Obesity also results in chronic, subclinical inflammation characterized by elevated circulating pro-inflammatory mediators [7, 8] that have been linked to progression [9] and may increase risk via altered adipokine levels and insulin resistance [10]. Locally, adipocyte hypertrophy occurs in the breast of overweight/obese women that can lead to adipocyte death, resulting in macrophage recruitment and inflamed white adipose tissue (WAT) [11]. This adipocyte-macrophage interaction is characterized by the encirclement of necrotic adipocytes by macrophages in a crown-like pattern [11]. These crown-like structures in the breast adipose tissue (CLS-B) create a unique microenvironment rich in pro-inflammatory cytokines. Among women with breast cancer, CLS-B are associated with NF-κB activation, increased aromatase activity, and elevation of pro-inflammatory mediators, which may enhance invasiveness and metastatic potential [12–16].

Previous epidemiologic studies have consistently demonstrated a strong, positive association between obesity, typically measured using body mass index (BMI), and CLS-B [16–22], although CLS-B are also reportedly present in up to one third of lean women (BMI < 25 kg/m^2^). These data suggest that factors other than obesity contribute to the formation of CLS-B [23], but few studies have examined associations for other characteristics such as race/ethnicity, smoking status, and reproductive history. One previous study of 150 Black, non-Black Latinas, and Caucasian women with breast cancer found the highest numbers of CLS in the tumor tissue of Black patients and subsequently found CLS to be associated with lower survival; however, BMI was not available in this study [24]. Another analysis of CLS-B with survival included a low proportion of women with CLS-B and few clinical outcomes [25]. A case-only analysis of 127 patients who all developed metastatic breast cancer found that CLS-B was associated with shorter time to recurrence [19]. Thus, large epidemiologic studies with detailed clinical information in multi-racial populations are needed to assess determinants of CLS-B and their association with clinical outcomes.

Accordingly, we examined whether other patient factors, independent of BMI, are associated with detection of CLS-B in normal adjacent breast WAT tissue of cancer patients and whether CLS-B are associated with progression-free and overall survival in a large multi-racial study population. Given that African-American women have a higher prevalence of obesity than White women and higher rates of aggressive breast cancer subtypes and mortality, we were particularly focused on comparing the prevalence and clinical impact of CLS-B among African-American versus White women. Thus, we examined the detection of CLS-B in a study population with approximately equal numbers of African-American (*n* = 174) and White patients (*n* = 168) with stage I–III breast cancer diagnosed and treated at Emory University hospitals.

## Methods

### Study population

**Fig. 1:**
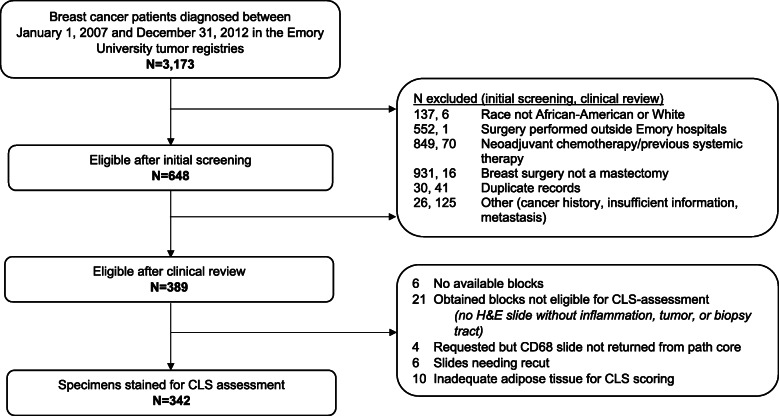
Flow chart illustrating patient selection and tissue availability

Women aged ≥ 18 years, diagnosed with primary invasive stage I–III breast cancer (ICD: C50) between January 1, 2007, and December 31, 2012, were identified via tumor registries associated with Emory University hospitals (*n* = 3173). Cases were initially screened and considered eligible if they were African-American or White, underwent mastectomy at an affiliated hospital, and had not received neoadjuvant treatment for their breast cancer or systemic therapy for the treatment of a previous cancer diagnosis (*n* = 648). Data abstractors further reviewed medical records for those passing initial screening and only included those meeting the above criteria upon review and clinical verification (*n* = 389). A further 47 patients were deemed ineligible upon specimen retrieval and staining due to unavailable tissue, inadequate adipose tissue for CLS scoring, or the presence of inflammation, tumor, or biopsy tract precluding accurate CLS-B assessment. Thus, 342 patients (*n* = 174 African-American, *n* = 168 White women) were included in final analyses (Fig. 1). Informed consent was provided by women undergoing mastectomy at Emory University hospitals. This study was approved by the Institutional Review Board of Emory University (00100602).

### Clinical data and biospecimen collection

Demographic and clinical characteristics were systematically abstracted by research staff using REDCap (Research Electronic Data Capture) tools hosted at Emory University [26, 27] and included information such as age at diagnosis, lifestyle factors (e.g., smoking history, alcohol intake), gynecologic and reproductive history (e.g., age at menarche, number of live births, menopausal status), medical conditions, family history of breast cancer among first-degree relatives, tumor characteristics (stage, grade, hormone receptor status, number of positive sentinel and axillary lymph nodes), and administered treatments. Independent data reviews were conducted for quality assurance. Menopausal status was categorized as premenopausal, perimenopausal, or postmenopausal based on clinical notes with women in the latter group exhibiting one of the following characteristics at the time of diagnosis: (1) having had bilateral oophorectomy, (2) reporting permanent cessation of menses for 12 or more months in the absence of chemotherapy or endocrine therapy, or (3) age > 55 years at diagnosis if data were missing. BMI was calculated using height and weight recorded prior to surgery (measured continuously as well as using cut-points based on World Health Organization (WHO) definitions: BMI < 25 kg/m^2^ [under or ideal weight], BMI 25–29.9 kg/m^2^ [overweight], or BMI ≥ 30 kg/m^2^ [obese]) [28]. Tumors were classified as estrogen receptor (ER) and/or progesterone receptor (PR) positive if > 1% staining by immunohistochemistry (IHC) was reported clinically.

Non-tumor containing breast WAT specimens were obtained under a standard tissue acquisition protocol. For each participant, up to three formalin-fixed, paraffin-embedded (FFPE) blocks of normal adjacent breast tissue from quadrants uninvolved by cancer were selected. Specimens were examined with hematoxylin and eosin (H&E) staining by a pathologist (AMC, UK, MES) to ensure samples were representative of benign breast tissue. Blocks with the greatest area of fat and no evidence of biopsy tract change, fat necrosis, or increased inflammation were chosen to ensure the selection of the one best WAT enriched block per patient.

### Tissue and CLS-B assessment

**Fig. 2:**
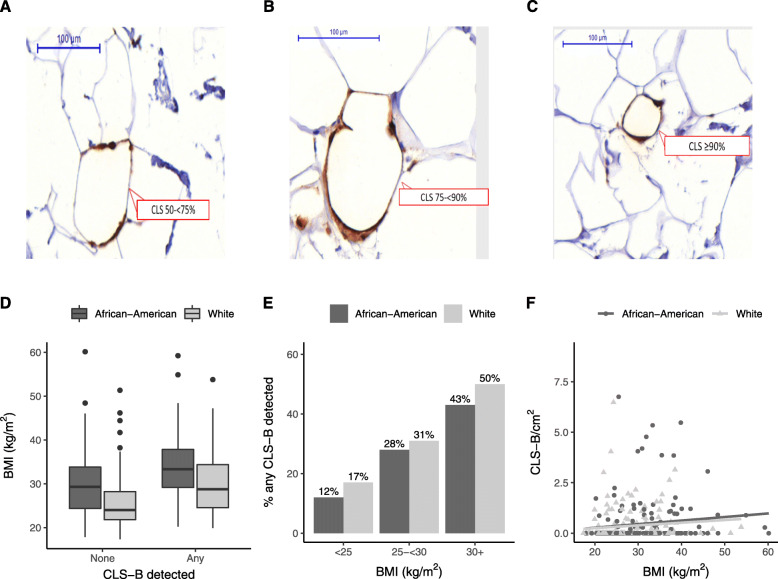
Analysis of crown-like structures in breast adipose tissue (CLS-B) among African-American and White breast cancer patients. **a** CD68-immunostained tissue showing 50 to < 75% adipocyte encirclement. **b** CD68-immunostained tissue showing 75 to < 90% adipocyte encirclement. **c** CD68-immunostained tissue showing ≥ 90% adipocyte encirclement. **d** BMI (kg/m^2^) distribution by CLS-B detection and race. **e** Percent of patients with any CLS-B by BMI category and race. **f** Spearman rank correlation between BMI (kg/m^2^) and CLS-B/cm^2^ (overall 0.27, *P* < 0.001, African-American 0.25, *P* = 0.001, White 0.29, *P* < 0.001)

To evaluate the presence of CLS-B, consistent with previously established methods [21], deparaffinized, rehydrated sections obtained from selected FFPE blocks were stained for CD68 using IHC (Dako Envision (Dako) automated system for detection of CD68; 1:200 dilution, monoclonal mouse anti-human CD68 clone KP1, M0814, DAKO, Denmark) at Winship Cancer Institute’s Pathology Core Laboratory (Emory University). From the corresponding tissue blocks, unstained tissue sections were sequentially cut within an estimated 100 to 200 μm of the original H&E-stained section. Whole slide digital images of anti-CD68-immunostained slides were captured with the 3DHISTECH Panoramic Scanner 150 and images analyzed using Panoramic Viewer 1.15.4 (3DHISTECH Ltd., Budapest, Hungary). Adipose tissue areas were defined as those containing ≥ 75% adipose, and total breast WAT area (cm^2^) was determined as the product of total fat percentage (visually estimated) and the total tissue area on the slide measured with 3DHISTECH software. The presence and number of CLS-B were assessed within the observed fat area on the whole tissue slide, and each CLS-B was manually counted and annotated on the digital image. Each CLS-B was defined according to the proportion of adipocyte encirclement by CD68-positive macrophages as 50 to < 75%, 75 to < 90%, and ≥ 90% (Fig. 2a–c). In the primary analyses, the presence of CLS-B (yes, no) was defined as detection of any CLS-B with ≥ 50% adipocyte encirclement. In supplementary analyses, we examined associations with any CLS-B detected using ≥ 75% and ≥ 90% adipocyte encirclement to classify presence. Reviewers were masked to clinicopathological data and outcomes at the time of scoring. CD68 and CLS-B were visually assessed by a single reviewer (AMC) with validation of a subset (*n* = 80) by two independent pathologists (UK and MES), with a Kappa score > 0.75 for both two-way comparisons. CLS-B density, quantified as the number of CLS-B per square centimeter of breast WAT (CLS-B/cm^2^), was estimated, and the median (0.87 CLS-B/cm^2^) was considered the cutoff to differentiate between low and high CLS-B density.

As a quality control measure, adipocyte number was examined in a subset of participants (*n* = 81) determined by counting the number of nuclei per 10X power fields. BMI was inversely associated with the number of adipocytes (Spearman rank correlation − 0.47, 95% CI = − 0.64, − 0.29, *P* < 0.001; Additional file 1: Supplementary Fig. 1A). CLS-B/cm^2^ was also negatively correlated with the number of adipocytes, although not as strongly as for BMI (Spearman rank correlation − 0.18, 95% CI = − 0.40, 0.07, *P* = 0.12; Additional file 1: Supplementary Fig. 1B).

### Statistical analysis

Selected characteristics of the overall study population, by race and by detection of CLS-B, were summarized by mean and standard deviation (SD) for continuous variables and by frequencies and percentages for categorical variables. Logistic regression models were used to determine odds ratios (ORs) and 95% confidence intervals (CIs) for associations between race, BMI, and other potential factors with the detection of CLS-B (yes, no). Proportional odds models were used to examine associations between potential risk factors and CLS-B density (none, low, high). When examining associations with tumor characteristics, CLS-B status was treated as the exposure and logistic regression was used when the outcome was a binary tumor characteristic (ER status or lymph node status) and proportional odds models for tumor characteristics such as stage and grade, which are ordinal in nature. Potential confounders were selected for inclusion in multivariable models based on known associations in the literature and causal graphical analyses (directed acyclic graphs, DAGs).

For analyses examining associations between CLS-B detection and clinical outcomes, overall survival (OS) time and progression-free survival (PFS) time were calculated from the date of diagnosis until the date of breast cancer recurrence (for PFS only), death, last follow-up, or December 31, 2018, whichever came first. Breast cancer recurrence was determined via clinical data abstraction using REDCap. Vital status was determined by the Georgia Center for Cancer Statistics via routine linkage with the state mortality file, the National Death Index, and through active follow-up/other administrative sources. We excluded 15 non-Georgia residents lacking follow-up data and 8 with missing covariate information from survival analyses. Survival outcomes by any CLS-B detection were examined using Kaplan-Meier curves and the log-rank test as well as using age-adjusted and multivariable Cox proportional hazards regression to compute hazard ratios (HRs) and 95% confidence intervals (CIs). Multivariable models adjusted for age at diagnosis (years), BMI (kg/m^2^), and smoking status (never, former, and current smokers) as these variables were determined to be associated with both CLS-B detection and clinical outcomes. The proportional hazards assumption was tested for each survival outcome using interaction terms between CLS-B exposure and the log of survival time. There was no violation for associations between CLS-B and overall survival. A violation was observed for the association between any CLS-B and PFS (*P* = 0.02); however, inspection of the log-log survival curves and Schoenfeld residuals revealed the violation was towards the end of the follow-up when few women remained under observation. Truncation at 6 years of follow-up did not materially change associations; thus, results including the full follow-up time are presented.

## Results

### Study population

The distribution of demographic and clinical characteristics at diagnosis is presented in Table 1. Mean age at breast cancer diagnosis was 56 years (SD 13.9; range 24–92) among African-American women and 55 years (SD 12.4 years; range 30–85) among White women. Approximately 63% were postmenopausal in both groups; however, African-American women were more likely to experience menopause at < 45 years of age (54% vs 38%) with a slightly higher proportion of surgical menopause (43% vs 38%). Mean BMI was 31.1 kg/m^2^ (SD 7.6) among African-American women and 26.9 kg/m^2^ (SD 6.5) among White women. African-American women were more likely than White women to have ER− tumors (30% vs 12%), to have poorly differentiated tumors (41% vs 22%), and to receive adjuvant chemotherapy (57% vs 39%).

**Table 1:** Demographic and clinical characteristics of 342 women diagnosed with invasive breast cancer (stages I–III) by race, Emory University, 2007–2012

		Race	
	Total	African-American	White
	(***N*** = 342)	(***N*** = 174)	(***N*** = 168)
***Demographic characteristics***			
**Age at diagnosis (years)**			
Mean (SD)	55.25 (13.20)	55.71 (13.91)	54.78 (12.43)
**BMI (kg/m**^**2**^**),*n*(%)**			
< 25	126 (37.1)	40 (23.3)	86 (51.2)
25 to < 30	85 (25.0)	43 (25.0)	42 (25.0)
≥ 30	129 (37.9)	89 (51.7)	40 (23.8)
**Smoking status,*n*(%)**			
Non-smoker	225 (67.0)	111 (64.5)	114 (69.5)
Past smoker	87 (25.9)	44 (25.6)	43 (26.2)
Current smoker	24 (7.1)	17 (9.9)	7 (4.3)
**Age at menarche (years)**			
Mean (SD)	12.68 (1.66)	12.59 (1.69)	12.77 (1.63)
**Parity**			
Nulliparous	44 (15.0)	19 (13.1)	25 (16.9)
Parous (1+ live births)	249 (85.0)	126 (86.9)	123 (83.1)
**History of breastfeeding**^**a**^**,*n*(%)**			
No	75 (39.5)	38 (45.2)	37 (34.9)
Yes	115 (60.5)	46 (54.8)	69 (65.1)
**Menopausal status,*n*(%)**			
Pre-/perimenopausal	117 (36.1)	59 (36.9)	58 (35.4)
Postmenopausal	207 (63.9)	101 (63.1)	106 (64.6)
**Age at menopause**^**b**^**,*n*(%)**			
< 45 years	83 (45.4)	46 (53.5)	37 (38.1)
45 to < 50 years	35 (19.1)	17 (19.8)	18 (18.6)
50 to < 55 years	50 (27.3)	17 (19.8)	33 (34.0)
≥ 55 years	15 (8.2)	6 (7.0)	9 (9.3)
**History of hormone replacement therapy use**^**b**^**,*n*(%)**			
No	120 (63.8)	66 (75.0)	54 (54.0)
Yes	68 (36.2)	22 (25.0)	46 (46.0)
**Family history of breast cancer among first-degree relatives,*n*(%)**			
No	240 (73.6)	118 (74.2)	122 (73.1)
Yes	86 (26.4)	41 (25.8)	45 (26.9)
**Diabetes status,*n*(%)**			
No	298 (87.1)	140 (80.5)	158 (94.0)
Yes	44 (12.9)	34 (19.5)	10 (6.0)
**Hypertension status,*n*(%)**			
No	200 (58.5)	82 (47.1)	118 (70.2)
Yes	142 (41.5)	92 (52.9)	50 (29.8)
**High cholesterol status,*n*(%)**			
No	286 (83.6)	141 (81.0)	145 (86.3)
Yes	56 (16.4)	33 (19.0)	23 (13.7)
***Clinical characteristics***			
**ER status,*n*(%)**			
Positive	267 (79.0)	120 (70.2)	147 (88.0)
Negative	71 (21.0)	51 (29.8)	20 (12.0)
**Stage,*n*(%)**			
Stage I	163 (47.7)	76 (43.7)	87 (51.8)
Stage II	135 (39.5)	75 (43.1)	60 (35.7)
Stage III	44 (12.9)	23 (13.2)	21 (12.5)
**Tumor grade,*n*(%)**			
Well differentiated	75 (22.9)	30 (18.2)	45 (27.6)
Moderately differentiated	150 (45.7)	68 (41.2)	82 (50.3)
Poorly differentiated	103 (31.4)	67 (40.6)	36 (22.1)
**Tumor size (cm),*n*(%)**			
≤ 0.5	7 (2.4)	2 (1.4)	5 (3.3)
0.5 to < 1	43 (14.5)	20 (13.9)	23 (15.0)
1 to < 5	222 (74.7)	102 (70.8)	120 (78.4)
≥ 5	25 (8.4)	20 (13.9)	5 (3.3)
**Number of positive lymph nodes,*n*(%)**			
0	204 (65.6)	101 (64.7)	103 (66.5)
≥ 1	107 (34.4)	55 (35.3)	52 (33.5)
**Chemotherapy,*n*(%)**			
No	172 (52.4)	70 (43.5)	102 (61.1)
Yes	156 (47.6)	91 (56.5)	65 (38.9)
**Radiation,*n*(%)**			
No	235 (71.4)	105 (65.2)	130 (77.4)
Yes	94 (28.6)	56 (34.8)	38 (22.6)
**Hormone therapy,*n*(%)**			
No	109 (31.9)	71 (40.8)	38 (22.6)
Yes	233 (68.1)	103 (59.2)	130 (77.4)

*Abbreviations*: *BMI* body mass index, *SD* standard deviation

Note: Missing values were not included in the calculation of percentages

^a^Among parous women

^b^Among postmenopausal women only

### CLS-B detection and density by patient characteristics at diagnosis

Detection of any CLS-B was similar between African-American (32%) and White (29%) patients with no evidence of an association between race and CLS-B in multivariable models adjusting for age at diagnosis and BMI (OR = 0.82, 95% CI = 0.49, 1.36) (Table 2). BMI was strongly and consistently associated with detection and density of CLS-B (Fig. 2d–f). As compared with under or ideal weight women, overweight women were more than twice as likely to have any detection of CLS-B (OR = 2.34, 95% CI = 1.17–4.70) and obese women more than four times as likely (OR = 4.73, 95% CI = 2.48–9.01), adjusting for age at diagnosis, race, and smoking status. As shown in Supplementary Table 1 in Additional file 1, BMI associations were similar for African-American women (*overweight*: OR = 2.60, 95% CI = 0.79–8.54; *obese*: OR = 5.28, 95% CI = 1.83–15.20) and White women (*overweight*: OR = 2.26, 95% CI = 0.93–5.50; *obese*: OR = 4.79, 95% CI = 2.01–11.50) with no evidence of effect modification by race (*P* = 0.97). Age at diagnosis was also positively associated with detection of any CLS-B (*≥ 60 vs < 50 years*: OR = 1.78, 95% CI = 0.99–3.21) and higher CLS-B density (OR = 1.85, 95% CI = 1.05–3.26). Parity was inversely associated with detection of any CLS-B with an OR of 0.49 (95% CI = 0.24–1.01) for parous women compared to nulliparous women. Current smoking was positively associated with detection of any CLS-B (OR = 2.13, 95% CI = 0.87–5.21) and higher CLS-B density (OR = 2.30, 95% CI = 0.99–5.32), compared to non-smokers; however, this pattern was not consistent across other CLS-B classification schemes likely due to the small number of current smokers (*n* = 24) in the study (Additional file 1: Supplementary Table 2). Associations for age at menarche, history of breastfeeding among parous women, menopausal status, hormone replacement therapy use among postmenopausal women, family history of breast cancer, diabetes, hypertension, and high cholesterol were null or too imprecise to make an inference.

**Table 2:** Associations of potential risk factors with the detection and density of crown-like structures in breast adipose tissue (CLS-B) among 342 women diagnosed with invasive breast cancer (stages I–III) at Emory University between 2007 and 2012

	Any CLS-B^**a**^				CLS-B Density (CLS-B/cm^**2**^)				
Characteristic	No ***N*** = 239	Yes ***N*** = 103	Unadjusted OR^**b**^ (95% CI)	Adjusted OR^**b**^ (95% CI)	None ***N*** = 239	Low (< 0.87) ***N*** = 51	High (≥ 0.87) ***N*** = 52	Unadjusted OR^**c**^ (95% CI)	Adjusted OR^**c**^ (95% CI)
	*N* (%)	*N* (%)			*N* (%)	*N* (%)	*N* (%)		
**Race**^**d**^									
White	120 (50)	48 (47)	1.00 (−)	1.00 (−)	120 (50)	28 (55)	20 (38)	1.00 (−)	1.00 (−)
African-American	119 (50)	55 (53)	1.16 (0.73–1.84)	0.82 (0.49–1.36)	119 (50)	23 (45)	32 (62)	1.23 (0.78–1.94)	0.95 (0.58–1.54)
**Age at diagnosis (years)**^**d**^									
25 to < 50	86 (36)	27 (26)	1.00 (−)	1.00 (−)	86 (36)	14 (27)	13 (25)	1.00 (−)	1.00 (−)
50 to < 60	74 (31)	30 (29)	1.29 (0.70–2.37)	1.20 (0.64–2.25)	74 (31)	17 (33)	13 (25)	1.26 (0.69–2.30)	1.19 (0.64–2.21)
≥ 60	79 (33)	46 (45)	1.85 (1.05–3.26)	1.78 (0.99–3.21)	79 (33)	20 (39)	26 (50)	1.89 (1.09–3.30)	1.85 (1.05–3.26)
**BMI (kg/m**^**2**^**)**^**d**^									
< 25	106 (45)	20 (19)	1.00 (−)	1.00 (−)	106 (45)	9 (18)	11 (21)	1.00 (−)	1.00 (−)
25 to < 30	60 (25)	25 (24)	2.21 (1.13–4.31)	2.34 (1.17–4.70)	60 (25)	14 (27)	11 (21)	2.13 (1.10–4.12)	2.19 (1.10–4.36)
≥ 30	71 (30)	58 (56)	4.33 (2.40–7.81)	4.73 (2.48–9.01)	71 (30)	28 (55)	30 (58)	4.16 (2.33–7.43)	4.48 (2.38–8.41)
**Smoking status**^**d**^									
Non-smoker	159 (67)	66 (66)	1.00 (−)	1.00 (−)	159 (67)	36 (72)	30 (60)	1.00 (−)	1.00 (−)
Past smoker	64 (27)	23 (23)	0.87 (0.50–1.51)	0.79 (0.44–1.41)	64 (27)	10 (20)	13 (26)	0.91 (0.52–1.56)	0.84 (0.48–1.48)
Current smoker	13 (6)	11 (11)	2.04 (0.87–4.78)	2.13 (0.87–5.21)	13 (6)	4 (8)	7 (14)	2.20 (0.97–4.97)	2.30 (0.99–5.32)
**Age at menarche**									
**(years)**^**d,e**^	12.7 (1.6)	12.7 (1.8)	0.98 (0.85–1.15)	1.06 (0.90–1.25)	12.7 (1.6)	12.6 (1.6)	12.7 (2.0)	0.99 (0.85–1.15)	1.06 (0.90–1.24)
**Parity**^**d**^									
Nulliparous	28 (13)	16 (19)	1.00 (−)	1.00 (−)	28 (13)	10 (23)	6 (15)	1.00 (−)	1.00 (−)
Parous (1+ live births)	181 (87)	68 (81)	0.66 (0.33–1.29)	0.49 (0.24–1.01)	181 (87)	34 (77)	34 (85)	0.71 (0.37–1.38)	0.57 (0.28–1.13)
**History of breastfeeding**^**d,f**^									
No	52 (38)	23 (44)	1.00 (−)	1.00 (−)	52 (38)	9 (35)	14 (54)	1.00 (−)	1.00 (−)
Yes	86 (62)	29 (56)	0.76 (0.40–1.46)	0.86 (0.44–1.70)	86 (62)	17 (65)	12 (46)	0.71 (0.38–1.35)	0.79 (0.41–1.52)
**Menopausal status**^**d**^									
Pre-/perimenopausal	89 (39)	28 (30)	1.00 (−)	1.00 (−)	89 (39)	14 (29)	14 (30)	1.00 (−)	1.00 (−)
Postmenopausal	140 (61)	67 (71)	1.52 (0.91–2.55)	1.41 (0.66–2.99)	140 (61)	35 (71)	32 (70)	1.49 (0.90–2.48)	1.46 (0.70–3.02)
**Age at menopause (years)**^**d,g**^									
< 45	55 (44)	28 (49)	1.00 (−)	1.00 (−)	55 (44)	11 (41)	17 (57)	1.00 (−)	1.00 (−)
45 to < 50	25 (20)	10 (18)	0.79 (0.33–1.86)	0.91 (0.38–2.21)	25 (20)	4 (15)	6 (20)	0.78 (0.34–1.82)	0.88 (0.37–2.08)
50 to < 55	37 (29)	13 (23)	0.69 (0.32–1.50)	0.74 (0.33–1.67)	37 (29)	8 (30)	5 (17)	0.64 (0.30–1.38)	0.67 (0.30–1.49)
≥ 55	9 (7)	6 (11)	1.31 (0.42–4.05)	1.13 (0.34–3.71)	9 (7)	4 (15)	2 (7)	1.11 (0.36–3.37)	0.91 (0.28–2.90)
**Hormone replacement therapy use**^**d,g**^									
No	77 (60)	43 (72)	1.00 (−)	1.00 (−)	77 (60)	25 (86)	18 (58)	1.00 (−)	1.00 (−)
Yes	51 (40)	17 (28)	0.60 (0.31–1.16)	0.74 (0.37–1.50)	51 (40)	4 (14)	13 (42)	0.69 (0.36–1.32)	0.87 (0.44–1.73)
**Family history of breast cancer**^**d**^									
No	165 (72)	75 (77)	1.00 (−)	1.00 (−)	165 (72)	38 (76)	37 (77)	1.00 (−)	1.00 (−)
Yes	63 (28)	23 (23)	0.80 (0.46–1.39)	0.80 (0.45–1.40)	63 (28)	12 (24)	11 (23)	0.80 (0.47–1.38)	0.82 (0.47–1.43)
**Diabetes mellitus**^**d**^									
No	210 (88)	88 (85)	1.00 (−)	1.00 (−)	210 (88)	43 (84)	45 (87)	1.00 (−)	1.00 (−)
Yes	29 (12)	15 (15)	1.23 (0.63–2.42)	0.82 (0.39–1.73)	29 (12)	8 (16)	7 (13)	1.20 (0.62–2.32)	0.80 (0.38–1.65)
**Hypertension**^**d**^									
No	144 (60)	56 (54)	1.00 (−)	1.00 (−)	144 (60)	27 (53)	29 (56)	1.00 (−)	1.00 (−)
Yes	95 (40)	47 (46)	1.27 (0.80–2.03)	0.86 (0.50–1.49)	95 (40)	24 (47)	23 (44)	1.25 (0.79–1.97)	0.88 (0.52–1.49)
**High cholesterol**^**d**^									
No	204 (85)	82 (80)	1.00 (−)	1.00 (−)	204 (85)	42 (82)	40 (77)	1.00 (−)	1.00 (−)
Yes	35 (15)	21 (20)	1.49 (0.82–2.72)	1.28 (0.68–2.42)	35 (15)	9 (18)	12 (23)	1.57 (0.88–2.82)	1.34 (0.73–2.47)

*Abbreviations*: *BMI* body mass index, *CI* confidence interval, *CLS-B* crown-like structures in the breast, *OR* odds ratio

Note: missing values were not included in the calculation of percentages

^a^Any CLS-B was defined as detection of ≥ 1 CLS-B with adipocyte encirclement ≥ 50% on the tissue section examined

^b^Estimated odds ratios using logistic regression models with any CLS-B (yes versus no) as the outcome

^c^Estimated odds ratios from proportional odds models; odds ratios represent the odds of increasing CLS-B (low or high CLS-B density versus no CLS-B; high CLS-B density versus none or low CLS-B density); score tests for the proportional odds assumption were not statistically significant (*P* > 0.05) for all models except for hormone replacement therapy use among postmenopausal women (*P* = 0.0032) which showed an inverse association for low CLS-B density (adjusted OR 0.28, 95% CI 0.09, 0.89) and a positive association for high CLS-B density (adjusted OR 1.47, 95% CI 0.66, 3.31) compared to no CLS-B

^d^All models were adjusted for age at diagnosis (years), race (African-American, White), and body mass index (kg/m^2^) except for those models where these covariates were the exposures of interest in which case covariates were mutually adjusted for; BMI models additionally adjusted for smoking status (never smoker, past smoker, current smoker)

^e^Values expressed as mean (standard deviation)

^f^Among parous women only

^g^Among postmenopausal women only

### Tumor characteristics and clinical outcomes by CLS-B detection and density

Detection of any CLS-B was not associated with any tumor characteristics examined including higher stage (OR = 0.95, 95% CI = 0.60–1.50), higher tumor grade (OR = 0.84, 95% CI = 0.53–1.34), ER− status (OR = 1.20, 95% CI = 0.65–2.19), or the presence of positive lymph nodes (OR = 1.05, 95% CI = 0.62–1.79), adjusting for age at diagnosis, race, and BMI in all models. The associations between CLS-B density and tumor characteristics were similar (Additional file 1: Supplementary Table 3).

**Fig. 3:**
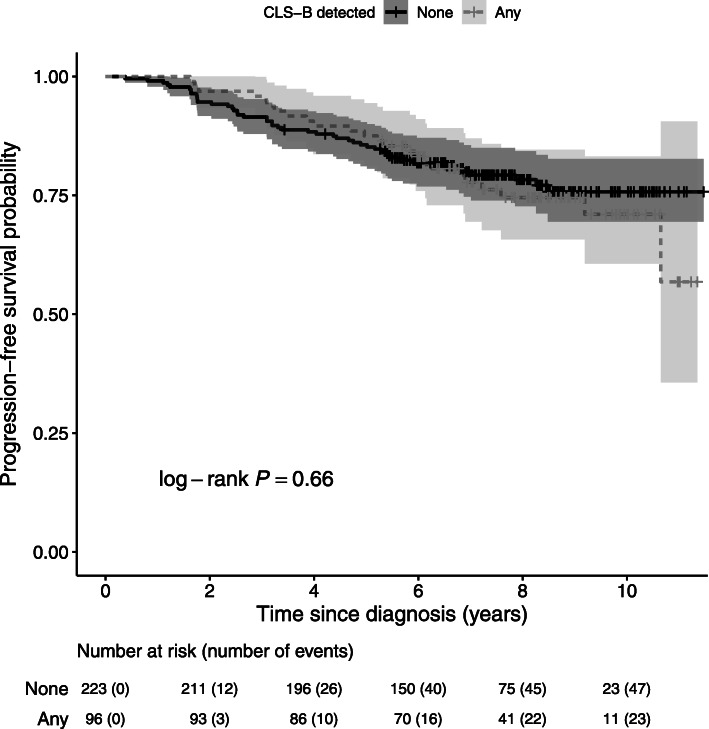
Kaplan-Meier curves comparing progression-free survival by detection of crown-like structures in breast adipose tissue (CLS-B)

Over a median of 8 years of follow-up, 46 breast cancer recurrences and 52 deaths (23 from breast cancer) occurred. The detection of any CLS-B was not associated with OS (HR = 1.02, 95% CI = 0.55–1.87) or PFS (HR = 0.99, 95% CI = 0.59–1.67), adjusting for age at diagnosis, BMI, and smoking status (Table 3; Fig. 3). Similar associations with OS and PFS were observed regardless of how CLS-B detection was determined (Additional file 1: Supplementary Table 4) or when CLS-B was further categorized by density (Table 3). When examined by race, we observed a difference in the direction of the association between any CLS-B and OS and PFS among African-American women (*OS*: adjusted HR = 1.18, 95% CI = 0.54–2.56; *PFS*: adjusted HR = 1.25, 95% CI = 0.64–2.46) compared to White women (*OS*: adjusted HR = 0.84, 95% CI = 0.30–2.30; *PFS*: adjusted HR = 0.75, 95% CI = 0.33–1.71), although the observed differences were not robust (*OS*: *P*_interaction_ = 0.75; *PFS*: *P*_interaction_ = 0.86).

**Table 3:** Associations between crown-like structures in breast adipose tissue (CLS-B) and clinical outcomes among 319 women diagnosed with invasive breast cancer (stages I–III) at Emory University between 2007 and 2012 with follow-up data through December 31, 2018

Exposure	Outcome	Category	Cases/person-years	Age-adjusted HR (95% CI)	Multivariable-adjusted HR (95% CI)
Any CLS-B^a,b^	Overall survival	No	32/1635	1.00 (−)	1.00 (−)
		Yes	18/726	1.12 (0.63–1.99)	1.02 (0.55–1.87)
CLS-B density (CLS-B/cm^2^)^b^		None	32/1635	1.00 (−)	1.00 (−)
		Low (< 0.87)	13/371	1.47 (0.77–2.82)	1.35 (0.68–2.68)
		High (≥ 0.87)	5/355	0.69 (0.27–1.76)	0.63 (0.24–1.65)
Any CLS-B^a,b^	Progression-free survival	No	47/1538	1.00 (−)	1.00 (−)
		Yes	24/704	1.05 (0.64–1.72)	0.99 (0.59–1.67)
CLS-B density (CLS-B/cm^2^)^b^		None	47/1538	1.00 (−)	1.00 (−)
		Low (< 0.87)	14/362	1.15 (0.63–2.09)	1.08 (0.58–2.02)
		High (≥ 0.87)	10/341	0.95 (0.48–1.88)	0.90 (0.45–1.81)

*Abbreviations*: *CI* confidence interval, *CLS-B* crown-like structures in the breast, *HR* hazards ratio

^a^Any CLS-B was defined as detection of ≥ 1 CLS-B with adipocyte encirclement ≥ 50% on the tissue section examined

^b^Adjusted for age at diagnosis (years), body mass index (kg/m^2^), and smoking status (never smoker, past smoker, current smoker)

### Assessment of technical sensitivity

Given that the presence of any CLS-B was determined using only one tissue section per case, the frequency of CLS-B is likely underestimated, although not differentially by patient characteristics or clinical outcomes—given that the pathologic review was blinded. This non-differential misclassification is expected to bias results towards the null and can be quantitatively assessed using bias analysis techniques (see Additional file 1: Supplementary Methods) [29]. For analyses examining associations between patient characteristics and detection of CLS-B, we performed a simple bias analysis assuming a sensitivity of 60% and a specificity of 99% that showed the unadjusted OR for the association between race and CLS-B would be 1.23 after accounting for misclassification of CLS-B which is similar to the observed OR and contained within the 95% CI (OR = 1.16, 95% CI 0.73–1.84). To quantify how much the observed HR for OS and PFS may have been biased towards the null, we conducted a probabilistic bias analysis (PBA) with 50,000 iterations which randomly sampled values of sensitivity and specificity at each iteration assuming trapezoidal distributions for sensitivity (min = 0.45, 0.55 ≤ mode ≤ 0.75, max = 0.8) and specificity (min = 0.9, 0.95 ≤ mode ≤ 0.99, max = 1.0) and then reconstructed the data that would have been observed had the misclassified variable been correctly classified [29]. Models included the same covariates as the primary analysis and incorporated both random error and error due to misclassification in the simulation interval (SI) [29]. Bias-adjusted estimates were HR = 1.29 (95% SI = 0.60–2.89) for OS and HR = 1.17 (95% SI = 0.61–2.26) for PFS.

## Discussion

The present study provides additional evidence that higher BMI is strongly associated with the detection and density of CLS-B [16–22] and provides new evidence that this association is similar in both White and African-American breast cancer patients. Race was not observed to be independently associated with detection or density of CLS-B. When we examined clinical outcomes over a median follow-up of 8 years, we found no difference in progression-free survival or overall survival by detection of CLS-B in unadjusted or adjusted models, contrary to two previous studies [19, 24]. However, estimates for these associations were imprecise, necessitating further research in larger study populations.

BMI is positively associated with CLS-B detection and density in numerous independent populations of breast cancer patients [16, 18, 20, 21, 24] and patients with benign breast disease [17]. The hypothesized biological mechanism relates higher BMI with adipocyte hypertrophy and the subsequent formation of CLS-B [5, 13]. Previous studies have found positive correlations between BMI, adipocyte size, and CLS-B that support this hypothesis [13, 18, 20]. Similarly, we found a positive correlation between BMI and CLS-B detection and density, and our finding of an inverse association between BMI and adipocytes per unit area is compatible with prior data linking obesity to adipocyte enlargement [18].

The association between BMI and CLS-B appears consistent across racial/ethnic groups as evidenced by the present study comparing African-American and White patients as well as previous studies in Taiwanese [20] and Hispanic/Latina [22] breast cancer patient populations. We did not find evidence that race is associated with the detection or density of CLS-B. The similar proportion of CLS-B+ cases among African-American (32%) and White women (29%) despite the higher prevalence of obesity among African-American women (52% vs 24%) was driven by the lower proportion of CLS-B+ cases at every BMI level compared to White women (Fig. 2e and Supplementary Table 1). For example, 43% of obese African-American women were CLS-B+ while 50% were CLS-B+ among obese White women. This may be a reflection of race-dependent differences in body composition since African-Americans are known to have lower overall adiposity and percent body fat than Whites with the same BMI [30]. However, BMI was still strongly associated with CLS-B among African-Americans and when adjusted for in analyses, any positive association between race and CLS-B attenuated considerably. Differences in BMI or other patient factors could explain the previous finding of an association between Black race and CLS-B [24].

Data on the association between other risk factors and detection or density of CLS-B are sparse. In the present study, age ≥ 60 years at diagnosis was related to increased detection of CLS-B. Previous studies have found that patients with CLS-B tend to be older than those without CLS-B [16, 21, 22, 25] although all but one study estimate included the null [25]. Menopausal status has also been identified as related to CLS-B [16]. Current smoking was also associated with increased detection of CLS-B in our study although estimates were imprecise and depended on the definition used for detection of CLS-B (i.e., ≥ 50%, ≥ 75%, or ≥ 90% adipocyte encirclement). One previous study in Hispanic/Latina breast cancer patients also observed a positive association with current smoking; however, this study and the present study had few current smokers (*n* = 8 in the study by Greenlee et al. [22] and *n* = 24 in the present study). We also observed an inverse association between parity and CLS-B detection, which has not been previously reported. Future studies are needed to confirm whether the risk factors identified in the present study persist in other, larger study populations—ideally with pre-malignant breast tissue.

We did not observe an association between CLS-B detection or density with overall or progression-free survival, contrary to two previous studies that found CLS-B to be associated with worse clinical outcomes among breast cancer patients [19, 24]. There are several differences in the methods for CLS-B evaluation and outcome assessment between our study and the previous ones that could account for the discrepant results. In the study by Koru-Sengul et al., CLS was assessed using three different macrophage markers for detection (CD206, CD40, and CD163), and the observed association with worse overall survival was limited to CLS detected by CD40, an M1 macrophage marker that has been linked to pro-inflammatory conditions [24]. It is possible that different types of macrophages (M1 vs M2) have different effects on clinical outcomes that could be obscured by using a pan-macrophage marker like CD68, which was employed in the present study. In further contrast to our study, CLS was analyzed using banked tumor tissue in the study by Koru-Sengul et al., and it was unclear how far the evaluated adipose tissue was from the tumor. We evaluated adipose tissue remote from the tumor to avoid local effects of biopsy or inflammatory responses directed against the cancer cells as opposed to localized adipose tissue specifically. In the study by Iyengar et al., CLS-B was detected by CD68 staining, which was the same as the present study, but assessed time to distant recurrence in a group of patients that all developed metastatic disease, limiting generalizability of their study findings [19]. In contrast, we evaluated patients unrestricted to outcome. Another key difference was that the previous study by Iyengar et al. used five tissue sections per case to determine the presence of CLS-B (41% CLS-B+) while the present study used one (30% CLS-B+), which could have resulted in more frequent misclassification of CLS-B presence in our study. We attempted to adjust for this potential misclassification using a probabilistic bias analysis with the results suggesting a potential bias towards the null in our main analysis but with a large amount of uncertainty—as demonstrated by the wide simulation interval (*PFS*: HR = 1.17, 95% SI 0.61–2.26). This estimate was still closer to the null than the estimate found in the study by Iyengar et al. (HR = 1.83, 95% CI = 1.07–3.13), so the discrepant results are likely due to other differences between the two studies [19]. For instance, our study population included equal proportions of African-American and White women while the Iyengar et al. study included predominantly White women. Heterogeneity in associations by race such that CLS-B increases risk in one group but decreases risk in the other (potentially driven by differences in tumor subtype by race) could result in an overall null association. Larger studies with enough statistical power to stratify by both race and tumor subtype are needed to examine these potential differences and further understand how CLS-B might influence breast cancer prognosis. Finally, it seems possible that if BMI and CLS-B are etiologically related to breast cancer development, then index event bias—a type of collider bias commonly referred to as the “obesity paradox”—could lead to artificial associations between CLS-B and prognosis [31]. Given the limitations of the previous studies and the uncertainty surrounding the estimates in the present study (i.e., wide 95% CIs), it remains unclear whether CLS-B negatively influences breast cancer prognosis and further research is needed.

The present study has several strengths and limitations. Strengths include the large number of well-characterized breast cancer patients with detailed follow-up information. Additionally, sampling equal numbers of African-American and White breast cancer patients allowed for comparisons by race. However, because our study population only consisted of breast cancer patients, our results do not provide evidence for how CLS-B may affect the risk and development of breast cancer. To date, there has only been one study that has examined the association between CLS-B and the risk of breast cancer that was among patients with benign breast disease and found a higher risk for patients with abundant CLS-B [17]. Further, our study was limited to mastectomy specimens to enable sampling of breast fat remote from the cancers, which may have resulted in some differences in patients versus those treated with lumpectomy. Additionally, the use of only one tissue specimen per case to assess CLS-B could have resulted in lower detection. This potential misclassification is expected to be non-differential since pathologists were blinded to patient characteristics and clinical outcomes at the time of CLS-B assessment. This non-differential misclassification is less likely to influence the analyses examining risk factors for CLS-B, supported by our simple bias analysis and the consistency of the strong association with BMI in our study with those from previous studies [18, 21]. Under-detection of CLS-B may have biased our results with OS and PFS towards the null; however, accounting for this potential misclassification using probabilistic bias analysis resulted in only weak to moderate bias-adjusted HRs for both (HR = 1.17 for PFS; HR = 1.29 for OS). Finally, while most previous studies have used CD68 for detection of CLS-B [16, 19–21], it does not distinguish between types of macrophages (M1 vs M2) that could be associated with risk factors and prognosis differently.

## Conclusions

This study further demonstrates the consistency and strength of association between BMI and detection and density of CLS-B in both African-American and White women diagnosed with breast cancer. Similar to previous studies, we did not find evidence that CLS-B was associated with tumor characteristics at diagnosis. However, in contrast to two previous investigations, we did not observe worse overall or progression-free survival among those with CLS-B present, raising questions related to differences in methodology in CLS-B assessment and whether CLS-B is in fact related to worse clinical outcomes.

## Supplementary information

**Additional file 1:** Supplementary Methods for Assessment of Technical Sensitivity; **Supplementary Fig. 1.** Associations between the number of adipocytes and (A) body mass index (kg/m^2^) and (B) CLS-B/cm^2^ in a subsample of breast cancer patients with breast adipose tissue obtained via mastectomy (*n* = 81); **Supplementary Table 1.** Associations between body mass index and detection of crown-like structures in the breast stratified by race among women diagnosed with invasive breast cancer (stage I-III), Emory University, 2007–2012; **Supplementary Table 2.** Associations of potential risk factors with detection of crown-like structures in the breast using different adipocyte encirclement cut-points for defining any CLS-B detection than the main analysis among women diagnosed with invasive breast cancer (stage I-III), Emory University, 2007–2012; **Supplementary Table 3.** Associations between detection and density of crown-like structures in breast adipose tissue (CLS-B) and tumor characteristics among women diagnosed with invasive breast cancer (stage I-III), Emory University, 2007–2012.; **Supplementary Table 4.** Associations between the detection of crown-like structures in breast adipose tissue (CLS-B) using different adipocyte encirclement cut-points and clinical outcomes among women diagnosed with invasive breast cancer (stage I-III) at Emory University between 2007 and 2012 and followed until December 31, 2018 (*n* = 319). **Description of data**: Additional file 1.docx includes a section of supplementary methods followed by one supplementary figure and four supplementary tables.

## Data Availability

The data used during the current study are available from the study PI on reasonable request and with approval of Emory’s IRB.

## Abbreviations

BMI: Body mass index
CI: Confidence interval
CLS-B: Crown-like structures in the breast
DAG: Directed acyclic graph
ER: Estrogen receptor
FFPE: Formalin-fixed paraffin-embedded
HR: Hazard ratio
H&E: Hematoxylin and eosin
IHC: Immunohistochemistry
OR: Odds ratio
OS: Overall survival
PBA: Probabilistic bias analysis
PFS: Progression-free survival
PR: Progesterone receptor
WAT: White adipose tissue
WHO: World Health Organization
