# Supplementary material for: Detection of crown-like structures in breast adipose tissue and clinical outcomes among African-American and White women with breast cancer
Source: Breast Cancer Res. 2020 Jun 17;22:65. doi: 10.1186/s13058-020-01308-4 (PMC7298873; doi:10.1186/s13058-020-01308-4)
Supplement: Supplementary file 1 — Additional file 1: Supplementary Methods for Assessment of Technical Sensitivity; Supplementary Fig. 1. Associations between the number of adipocytes and (A) body mass index (kg/m2) and (B) CLS-B/cm2 in a subsample of breast cancer patients with breast adipose tissue obtained via mastectomy (n = 81); Supplementary Table 1. Associations between body mass index and detection of crown-like structures in the breast stratified by race among women diagnosed with invasive breast cancer (stage I-III), Emory University, 2007–2012; Supplementary Table 2. Associations of potential risk factors with detection of crown-like structures in the breast using different adipocyte encirclement cut-points for defining any CLS-B detection than the main analysis among women diagnosed with invasive breast cancer (stage I-III), Emory University, 2007–2012; Supplementary Table 3. Associations between detection and density of crown-like structures in breast adipose tissue (CLS-B) and tumor characteristics among women diagnosed with invasive breast cancer (stage I-III), Emory University, 2007–2012.; Supplementary Table 4. Associations between the detection of crown-like structures in breast adipose tissue (CLS-B) using different adipocyte encirclement cut-points and clinical outcomes among women diagnosed with invasive breast cancer (stage I-III) at Emory University between 2007 and 2012 and followed until December 31, 2018 (n = 319). Description of data: Additional file 1.docx includes a section of supplementary methods followed by one supplementary figure and four supplementary tables. [file 13058_2020_1308_MOESM1_ESM.docx]

Additional File 1

**Supplementary Methods for Assessment of Technical Sensitivity**

Given that the presence of any CLS-B was determined using only one breast adipose tissue sample per case, it is possible that women truly with CLS-B were misclassified as not having CLS-B in our analysis. However, we do not believe this potential misclassification considerably influenced our results and conclusions for either 1) analyses examining selected clinical and demographic features in relation to CLS-B or 2) analyses examining CLS-B and PFS or OS. To demonstrate this, we conducted a series of bias analyses to quantify the potential bias from CLS-B misclassification.

1. **Analyses examining selected clinical and demographic features in relation to CLS-B**

In these analyses, CLS-B (yes or no) is the outcome. For the simple bias analysis, we assumed that specificity is very high (few false positives) and that sensitivity is likely lower (many false negatives). We also assumed that this misclassification did not differ by any of the clinical or demographic features since pathologists were blinded to patient characteristics during CLS-B assessment. If these assumptions are correct then results where false negatives are correctly classified as positives will be similar to our original analyses due to the fact that high specificity for outcome classification is often sufficient to obtain a valid ratio estimate even with poor sensitivity (1).

For example, the table on the left shows the observed crude table for examining the association between race and CLS-B. The table on the right has been "corrected" (in quotes since the truth cannot be known) for misclassification of CLS-B assuming non-differential specificiy (SP) and sensitivity (SE) [SP_E+_=SP_E-_=99% and SE_E+_=SE_E-_= 60%] using the equations from Lash et al. (1) in a simple bias analysis.

| \|  \| **Observed Data** \| \| \| \|  \| \| --- \| --- \| --- \| --- \| --- \| --- \| \|  \| **AA** \| \| **White** \| \| **Total** \| \| **CLS-B +** \| **55** \| **a** \| **48** \| **b** \| **103** \| \|  \|  \| \| **CLS-B -** \| **119** \| **c** \| **120** \| **d** \| **239** \| \|  \|  \| \| **Total** \| **174** \| **E+** \| **168** \| **E-** \|  \| \|  \|  \|  \| | \|  \| **Corrected Data** \| \| \| \|  \| \| --- \| --- \| --- \| --- \| --- \| --- \| \|  \| **AA** \| \| **White** \| \| **Total** \| \| **CLS-B +** \| **90.3** \| **A** \| **78.5** \| **B** \| **168.8** \| \|  \|  \| \| **CLS-B -** \| **83.7** \| **C** \| **89.5** \| **D** \| **173.2** \| \|  \|  \| \| **Total** \| **174** \| **E+** \| **168** \| **E-** \|  \| \|  \|  \|  \| |
| --- | --- | --- | --- | --- | --- | --- | --- | --- | --- | --- | --- | --- | --- | --- | --- | --- | --- | --- | --- | --- | --- | --- | --- | --- | --- | --- | --- | --- | --- | --- | --- | --- | --- | --- | --- | --- | --- | --- | --- | --- | --- | --- | --- | --- | --- | --- | --- | --- | --- | --- | --- | --- | --- | --- | --- | --- | --- | --- | --- | --- | --- | --- | --- | --- | --- | --- | --- | --- | --- | --- | --- | --- | --- | --- | --- |
| Crude OR=1.16 (0.73-1.84) | Corrected OR=1.23 |

AA=African-American Bias analysis equations:

| A = [a – E+(1 - SP_E+_) ] / [SE_E+_ - (1 - SP_E+_)] | C = E+ - A |
| --- | --- |
| B = [b – E- (1 - SP_E-_) ] / [SE_E-_ - (1 - SP_E-_)] | D = E- - B |

The corrected OR (1.23) is not considerably different than the crude OR (1.16) observed in our study and is contained within the 95% CI of the crude OR. In fact, the sensitivity would have to be ≤36% for the corrected OR to be greater than the upper CI limit (≥1.84). Notably, assuming Se=60% results in corrected data that suggest an overall prevalence of CLS-B+ = 169/342 = 49%, which is similar to studies that have used 5 samples per case to determine CLS-B status (2-7). A sensitivity of 36% would result in corrected data suggesting an overall prevalence of CLS-B+ = 285/342 = 83%. This high of a prevalence seems unlikely unless we consider that nearly all women could have some CLS-B and prevalence will inevitably increase with the amount of adipose tissue assessed. Such a presumption would bring into question the utility of such a marker at least on the basis of classifying women as CLS-B+ or CLS-B-.

1. **Analyses examining CLS-B and PFS or OS**

In these analyses, CLS-B is the exposure. Again, we can assume that specificity is very high and sensitivity much lower. We can also assume that misclassification does not differ by clinical outcome status since pathologists were blinded to outcome information upon assessment. This non-differential misclassification would be expected to bias results towards the null (8). In an attempt to quantify the extent to which exposure misclassification biased the association between CLS-B and clinical outcomes, we conducted a probabilistic bias analysis (PBA) with 50,000 iterations which randomly sampled values of sensitivity and specificity at each iteration assuming trapezoidal distributions for sensitivity (min=0.45, 0.55≤ mode ≤0.75, max=0.8) and specificity (min=0.9, 0.95≤ mode ≤0.99, max=1.0) then reconstructed the data that would have been observed had the misclassified variable been correctly classified.(8) These sensitivity values were chosen based on results from a small pilot study conducted by Morris et al.(9) that found 14/30 patients had CLS-B present on at least one of five specimens examined with 7/14 CLS-B+ on 1/5 slides, 2/14 CLS-B+ each on 2/5 slides, 3/5 slides, and 4/5 slides, and 1/14 CLS-B+ on 5/5 slides. If one section from the five possible was randomly sampled per case, six cases on average would be classified as CLS-B+ out of the truly 14 CLS-B+ cases, resulting in a sensitivity of 43%. The pilot study also demonstrated that sensitivity increased with higher BMI; thus, we expect our study population, which has a high median BMI, to have a higher sensitivity than that observed in the pilot study. We expect that specificity is close to 100%, with false positives likely only occurring due to pathologist error.

As an example of one possible iteration of the process where sensitivity was randomly sampled as 61% and specificity was randomly sampled as 97%, the bias-adjusted HR for overall survival by detection of CLS-B based on the reconstructed data was 1.30 as compared to the observed HR of 1.02:

| **Data** | **Exposure** | **Category** | **Cases/Person-years** | **Multivariable-adjusted HR** |
| --- | --- | --- | --- | --- |
| Observed^1,2^ | Any CLS-B^1,2^ | No | 32 / 1,635 | 1.00 (referent) |
|  |  | Yes | 18 / 726 | 1.02 |
|  |  |  |  |  |
| Reconstructed^2,3^ | Any CLS-B^1,2^ | No | 21 / 1,223 | 1.00 (referent) |
|  |  | Yes | 30 / 1,138 | 1.30 |

^1^Data used for the primary analysis, which assumes perfect classification of any CLS-B (100% sensitivity and 100% specificity)

^2^Adjusted for the following covariates: age at diagnosis (years), body mass index (kg/m2), and smoking status (never smoker, past smoker, current smoker)

^3^Example of reconstructed data assuming sensitivity=61% and specificity=97% for the detection of crown-like structures in breast adipose tissue (CLS-B)

Because no single iteration (reconstructed data set) is assuredly correctly bias-adjusted, we repeated this process of reconstructing the data based on randomly sampled sensitivity and specificity values from the assumed distributions for each (described above) 50,000 times resulting in 50,000 bias-adjusted estimates. The median of these estimates is reported as the bias-adjusted estimate along with the 2.5^th^ percentile and 97.5^th^ percentile as the 95% simulation interval (SI) to reflect the uncertainty in the bias model. The 95% SI reported additionally accounts for random error using the approach described by Fox et al. (8). Adjusting for the same potential confounders as the main analyses, the results of the probabilistic bias analysis were a bias-adjusted HR=1.29 (95% SI: 0.60, 2.89) for overall survival and a bias-adjusted HR=1.17 (95% SI: 0.61, 2.26) for progression-free survival.

**Supplementary Results**

**Supplementary Figure 1.** Associations between the number of adipocytes and (A) body mass index (kg/m^2^) and (B) CLS-B/cm^2^ in a subsample of breast cancer patients with breast adipose tissue obtained via mastectomy (n=81)^1^

**
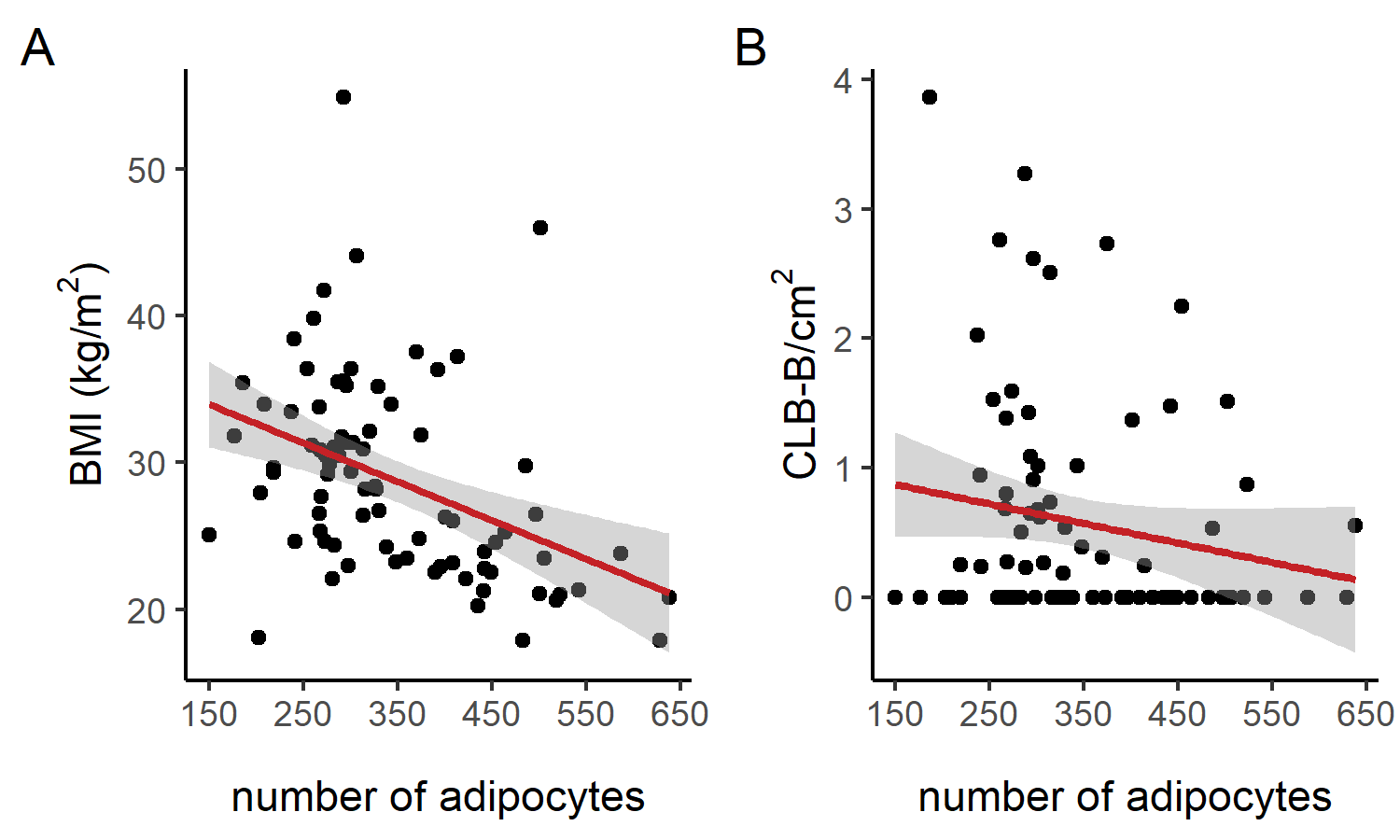
**

^1^Spearman rank correlation = -0.47 (95% CI: -0.64, -0.29), *P* < 0.001

^2^Spearman rank correlation = -0.18 (95% CI: -0.40, 0.07), *P* = 0.12

**Supplementary Table 1.** Associations between body mass index and detection of crown-like structures in the breast stratified by race among women diagnosed with invasive breast cancer (stage I-III), Emory University, 2007-2012

|  | **Any CLS-B** | |  |  |
| --- | --- | --- | --- | --- |
| **BMI (kg/m^2^)**  **African-American** | **No**  **N (%)** | **Yes**  **N (%)** | **Unadjusted OR**  **(95% CI)** | **Adjusted^1^ OR**  **(95% CI)** |
| <25 | 35 (30) | 5 (9) | 1.00 (-) | 1.00 (-) |
| 25-<30 | 31 (27) | 12 (22) | 2.57 (0.80, 8.22) | 2.60 (0.79, 8.54) |
| ≥30 | 51 (43) | 38 (69) | 5.21 (1.87, 14.60) | 5.28 (1.83, 15.20) |
| **White** |  |  |  |  |
| <25 | 71 (59) | 15 (31) | 1.00 (-) | 1.00 (-) |
| 25-<30 | 29 (24) | 13 (27) | 2.21 (0.93, 5.28) | 2.26 (0.93, 5.50) |
| ≥30 | 20 (17) | 20 (42) | 4.68 (2.00, 11.00) | 4.79 (2.01, 11.50) |

Note: missing values (n=2 missing BMI among African-American women) were not included in the calculation of percentages

^1^Adjusted for age at diagnosis, race, and smoking status

**Supplementary Table 2**. Associations of potential risk factors with detection of crown-like structures in the breast using different adipocyte encirclement cut-points for defining any CLS-B detection than the main analysis among women diagnosed with invasive breast cancer (stage I-III), Emory University, 2007-2012

|  | **Any CLS-B (**≥**75% adipocyte encirclement)^1^** | | | | **Any CLS-B (≥90% adipocyte encirclement)^2^** | | | | |
| --- | --- | --- | --- | --- | --- | --- | --- | --- | --- |
| **Characteristic** | **No**  **N (%)** | **Yes**  **N (%)** | **Unadjusted OR (95% CI)** | **Adjusted OR (95% CI)** | **No**  **N (%)** | **Yes**  **N (%)** | **Unadjusted OR (95% CI)** | **Adjusted OR (95% CI)** |  |
| **Race^3^** |  |  |  |  |  |  |  |  |  |
| Caucasian | 129 (49) | 39 (48) | 1.00 (-) | 1.00 (-) | 131 (49) | 37 (49) | 1.00 (-) | 1.00 (-) |  |
| African-American | 132 (51) | 42 (52) | 1.05 (0.64, 1.73) | 0.75 (0.44, 1.29) | 136 (51) | 38 (51) | 0.99 (0.59, 1.65) | 0.75 (0.43, 1.29) |  |
| **Age at diagnosis (years)^3^** |  |  |  |  |  |  |  |  |  |
| 25-<50 | 92 (35) | 21 (26) | 1.00 (-) | 1.00 (-) | 93 (35) | 20 (27) | 1.00 (-) | 1.00 (-) |  |
| 50-<60 | 81 (31) | 23 (28) | 1.24 (0.64, 2.41) | 1.15 (0.58, 2.27) | 82 (31) | 22 (29) | 1.25 (0.64, 2.45) | 1.16 (0.58, 2.31) |  |
| ≥60 | 88 (34) | 37 (46) | 1.84 (1.00, 3.39) | 1.78 (0.95, 3.35) | 92 (34) | 33 (44) | 1.67 (0.89, 3.12) | 1.61 (0.85, 3.06) |  |
| **BMI (kg/m^2^)^3^** |  |  |  |  |  |  |  |  |  |
| <25 | 110 (43) | 16 (20) | 1.00 (-) | 1.00 (-) | 110 (41) | 16 (21) | 1.00 (-) | 1.00 (-) |  |
| 25-<30 | 66 (26) | 19 (24) | 1.98 (0.95, 4.11) | 2.11 (0.98, 4.55) | 66 (25) | 19 (25) | 1.98 (0.95, 4.11) | 2.11 (0.98, 4.54) |  |
| ≥30 | 83 (32) | 46 (57) | 3.81 (2.02, 7.20) | 4.36 (2.17, 8.76) | 89 (34) | 40 (53) | 3.09 (1.62, 5.88) | 3.51 (1.74, 7.10) |  |
| **Smoking status^3^** |  |  |  |  |  |  |  |  |  |
| Non-Smoker | 170 (66) | 55 (71) | 1.00 (-) | 1.00 (-) | 175 (66) | 50 (69) | 1.00 (-) | 1.00 (-) |  |
| Past Smoker | 69 (27) | 18 (23) | 0.81 (0.44, 1.47) | 0.73 (0.39, 1.37) | 70 (27) | 17 (24) | 0.85 (0.46, 1.57) | 0.79 (0.42, 1.49) |  |
| Current Smoker | 19 (7) | 5 (6) | 0.81 (0.29, 2.28) | 0.81 (0.28, 2.34) | 19 (7) | 5 (7) | 0.92 (0.33, 2.59) | 0.93 (0.32, 2.69) |  |
| **Mean age at menarche** |  |  |  |  |  |  |  |  |  |
| **(years)^3^, (SD)** | 12.7 (1.6) | 12.6 (1.8) | 0.97 (0.82, 1.14) | 1.04 (0.88, 1.24) | 12.7 (1.7) | 12.6 (1.7) | 0.94 (0.79, 1.11) | 1.00 (0.83, 1.19) |  |
| **Parity^3^** |  |  |  |  |  |  |  |  |  |
| Nulliparous | 30 (13) | 14 (21) | 1.00 (-) | 1.00 (-) | 32 (14) | 12 (20) | 1.00 (-) | 1.00 (-) |  |
| Parous (1+ live births) | 197 (87) | 52 (79) | 0.57 (0.28, 1.14) | 0.43 (0.21, 0.91) | 200 (86) | 49 (80) | 0.65 (0.31, 1.36) | 0.54 (0.25, 1.15) |  |
| **History of** |  |  |  |  |  |  |  |  |  |
| **Breastfeeding^3,4^** |  |  |  |  |  |  |  |  |  |
| No | 59 (39) | 16 (41) | 1.00 (-) | 1.00 (-) | 60 (39) | 15 (41) | 1.00 (-) | 1.00 (-) |  |
| Yes | 92 (61) | 23 (59) | 0.92 (0.45, 1.89) | 1.00 (0.48, 2.10) | 93 (61) | 22 (59) | 0.95 (0.46, 1.97) | 1.01 (0.47, 2.14) |  |
| **Menopausal status^3^** |  |  |  |  |  |  |  |  |  |
| Pre/perimenopausal | 94 (38) | 23 (31) | 1.00 (-) | 1.00 (-) | 96 (37) | 21 (31) | 1.00 (-) | 1.00 (-) |  |
| Postmenopausal | 156 (62) | 51 (69) | 1.34 (0.77, 2.33) | 1.05 (0.47, 2.36) | 160 (63) | 47 (69) | 1.34 (0.76, 2.38) | 1.21 (0.53, 2.78) |  |
| **Age at menopause (years)^3,5^** |  |  |  |  |  |  |  |  |  |
| <45 | 63 (45) | 20 (46) | 1.00 (-) | 1.00 (-) | 64 (45) | 19 (46) | 1.00 (-) | 1.00 (-) |  |
| 45-<50 | 27 (19) | 8 (18) | 0.93 (0.37, 2.38) | 1.06 (0.41, 2.76) | 28 (20) | 7 (17) | 0.84 (0.32, 2.23) | 0.93 (0.34, 2.49) |  |
| 50-<55 | 38 (27) | 12 (27) | 0.99 (0.44, 2.26) | 1.04 (0.44, 2.43) | 39 (27) | 11 (27) | 0.95 (0.41, 2.21) | 0.95 (0.40, 2.27) |  |
| ≥55 | 11 (8) | 4 (9) | 1.15 (0.33, 4.00) | 0.95 (0.26, 3.50) | 11 (8) | 4 (10) | 1.22 (0.35, 4.29) | 1.02 (0.28, 3.72) |  |
| **Hormone replacement** |  |  |  |  |  |  |  |  |  |
| **therapy use^3,5^** |  |  |  |  |  |  |  |  |  |
| No | 86 (61) | 34 (72) | 1.00 (-) | 1.00 (-) | 89 (61) | 31 (72) | 1.00 (-) | 1.00 (-) |  |
| Yes | 55 (39) | 13 (28) | 0.60 (0.29, 1.23) | 0.71 (0.33, 1.53) | 56 (39) | 12 (28) | 0.62 (0.29, 1.30) | 0.71 (0.32, 1.55) |  |
| **Family history of** |  |  |  |  |  |  |  |  |  |
| **breast cancer^3^** |  |  |  |  |  |  |  |  |  |
| No | 182 (73) | 58 (74) | 1.00 (-) | 1.00 (-) | 188 (74) | 52 (72) | 1.00 (-) | 1.00 (-) |  |
| Yes | 66 (27) | 20 (26) | 0.95 (0.53, 1.70) | 0.96 (0.53, 1.75) | 66 (26) | 20 (28) | 1.10 (0.61, 1.97) | 1.11 (0.61, 2.02) |  |
| **Diabetes mellitus^3^** |  |  |  |  |  |  |  |  |  |
| No | 228 (87) | 70 (86) | 1.00 (-) | 1.00 (-) | 234 (88) | 64 (85) | 1.00 (-) | 1.00 (-) |  |
| Yes | 33 (13) | 11 (14) | 1.09 (0.52, 2.26) | 0.73 (0.32, 1.64) | 33 (12) | 11 (15) | 1.22 (0.58, 2.54) | 0.92 (0.41, 2.06) |  |
| **Hypertension^3^** |  |  |  |  |  |  |  |  |  |
| No | 157 (60) | 43 (53) | 1.00 (-) | 1.00 (-) | 160 (60) | 40 (53) | 1.00 (-) | 1.00 (-) |  |
| Yes | 104 (40) | 38 (47) | 1.33 (0.81, 2.20) | 0.96 (0.54, 1.72) | 107 (40) | 35 (47) | 1.31 (0.78, 2.19) | 1.02 (0.57, 1.84) |  |
| **High cholesterol^3^** |  |  |  |  |  |  |  |  |  |
| No | 221 (85) | 65 (80) | 1.00 (-) | 1.00 (-) | 225 (84) | 61 (81) | 1.00 (-) | 1.00 (-) |  |
| Yes | 40 (15) | 16 (20) | 1.36 (0.72, 2.59) | 1.16 (0.59, 2.35) | 42 (16) | 14 (19) | 1.23 (0.63, 2.40) | 1.08 (0.54, 2.16) |  |

Abbreviations: BMI, body mass index; CI, confidence interval; CLS-B, crown-like structures in the breast; OR, odds ratio

Note: missing values not included in the calculation of percentages

^1^ Any CLS-B defined as detection of ≥1 CLS-B with adipocyte encirclement ≥75% on the tissue section examined

^2^ Any CLS-B defined as detection of ≥1 CLS-B with adipocyte encirclement ≥90% on the tissue section examined

^3^ All models adjusted for age at diagnosis (years), race (African-American, White), and body mass index (kg/m^2^) except for those models where these covariates were the exposures of interest in which case covariates were mutually adjusted for; BMI models additionally adjusted for smoking status (never smoker, past smoker, current smoker)

^4^ Among parous women only

^5^ Among postmenopausal women only

**Supplementary Table 3**. Associations between detection and density of crown-like structures in breast adipose tissue (CLS-B) and tumor characteristics among women diagnosed with invasive breast cancer (stage I-III), Emory University, 2007-2012.

|  | **CLS-B Detection** | | **CLS-B Density (CLS-B/cm^2^)** | | | | | | |
| --- | --- | --- | --- | --- | --- | --- | --- | --- | --- |
| **Tumor characteristic** | **None**  **N (%)** | **Any^1^**  **N (%)** | **None**  **(0)**  **N (%)** | | | **Low**  **(>0-<0.87)**  **N (%)** | | **High**  **(0.87-36.8)**  **N (%)** | |
| ***Frequencies*** | ***N=239*** | ***N=103*** | ***N=239*** | |  | ***N=51*** | | ***N=52*** | |
| **ER status** |  |  |  | | |  | |  | |
| Positive | 192 (80) | 75 (76) | 192 (80) | | | 38 (76) | | 37 (76) | |
| Negative | 47 (20) | 24 (24) | 47 (20) | | | 12 (24) | | 12 (24) | |
| **Stage** |  |  |  | | |  | |  | |
| Stage I | 112 (47) | 51 (50) | 112 (47) | | | 24 (47) | | 27 (52) | |
| Stage II | 99 (41) | 36 (35) | 99 (41) | | | 20 (39) | | 16 (31) | |
| Stage III | 28 (12) | 16 (15) | 28 (12) | | | 7 (14) | | 9 (17) | |
| **Tumor grade^2^** |  |  |  | | |  | |  | |
| 1 | 51 (22) | 24 (25) | 51 (22) | | | 16 (34) | | 8 (16) | |
| 2 | 104 (45) | 46 (47) | 104 (45) | | | 20 (43) | | 26 (52) | |
| 3 | 76 (33) | 27 (28) | 76 (33) | | | 11 (23) | | 16 (32) | |
| **Number of positive lymph nodes** |  |  |  | | |  | |  | |
| 0 | 144 (66) | 60 (65) | 144 (66) | | | 27 (61) | | 33 (67) | |
| ≥1 | 74 (34) | 33 (35) | 74 (34) | | | 17 (39) | | 16 (33) | |
|  | **Any CLS-B**^1^ **vs None** | | **Low Density CLS-B vs None** | | | | **High Density CLS-B vs None** | | |
| ***Outcome*** | **Unadjusted OR**  **(95% CI)** | **Adjusted**  **OR (95% CI)** | **Unadjusted OR (95% CI)** | **Adjusted**  **OR (95% CI)** | | | **Unadjusted OR (95% CI)** | | **Adjusted**  **OR (95% CI)** |
| **ER- status^3,4^** | 1.29 (0.74, 2.26) | 1.20 (0.65, 2.19) | 1.31 (0.63, 2.71) | 1.13 (0.53, 2.42) | | | 1.28 (0.62, 2.63) | | 1.28 (0.59, 2.79) |
| **Higher stage^4,5^** | 1.00 (0.64, 1.55) | 0.95 (0.60, 1.50) | 0.95 (0.54, 1.69) | 0.89 (0.50, 1.60) | | | 1.04 (0.59, 1.85) | | 1.01 (0.55, 1.83) |
| **Higher tumor grade^2,4,5^** | 0.82 (0.53, 1.27) | 0.84 (0.53, 1.34) | 1.12 (0.63, 1.98) | 1.08 (0.60, 1.95) | | | 0.57 (0.32, 1.03) | | 0.62 (0.34, 1.15) |
| **≥1 positive lymph nodes^3,4^** | 1.08 (0.65, 1.81) | 1.05 (0.62, 1.79) | 0.96 (0.49, 1.85) | 0.93 (0.47, 1.82) | | | 1.24 (0.64, 2.42) | | 1.21 (0.60, 2.44) |

Abbreviations: CI, confidence interval; CLS-B, crown-like structures in the breast; OR, odds ratio

Note: missing values not included in the calculation of percentages

^1^ Any CLS-B was defined as detection of ≥1 CLS-B with adipocyte encirclement ≥50% on the tissue section examined

^2^ Tumor grade: 1=well differentiated, 2=moderately differentiated, and 3=poorly differentiated

^3^Estimated odds ratios using logistic regression models with the tumor characteristic of interest as the outcome

^4^Adjusted for age at diagnosis (years), race (African American, Caucasian), and body mass index (kg/m^2^)

^5^Estimated odds ratios using proportional odds regression models with the tumor characteristic of interest as the outcome and assuming the outcome is ordinal in nature; Score tests for the proportional odds assumption were not statistically significant (P >0.05) for all models

**Supplementary Table 4**. Associations between crown-like structures in breast adipose tissue (CLS-B) using different adipocyte encirclement cut-points and clinical outcomes among women diagnosed with invasive breast cancer (stage I-III) at Emory University between 2007 and 2012 and followed until December 31, 2018 (n=319)

| **Exposure** | **Outcome** | **Category** | **Cases/Person-years** | **Age-adjusted**  **HR (95% CI)** | **Multivariable-adjusted**  **HR (95% CI)** |
| --- | --- | --- | --- | --- | --- |
| Any CLS-B (≥75%)^,1,2^ | Overall survival | No | 38 / 1,799 | 1.00 (-) | 1.00 (-) |
|  |  | Yes | 12 / 562 | 0.90 (0.47, 1.73) | 0.82 (0.42, 1.60) |
|  |  |  |  |  |  |
| Any CLS-B (≥90%)^2,3^ |  | No | 39 / 1,848 | 1.00 (-) | 1.00 (-) |
|  |  | Yes | 11 / 513 | 0.94 (0.48, 1.83) | 0.87 (0.44, 1.72) |
| Any CLS-B (≥75%)^,1,2^ | Progression-free survival | No | 54 / 1,699 | 1.00 (-) | 1.00 (-) |
|  |  | Yes | 17 / 543 | 0.93 (0.54, 1.61) | 0.88 (0.50, 1.55) |
|  |  |  |  |  |  |
| Any CLS-B (≥90%)^2,3^ |  | No | 56 / 1,742 | 1.00 (-) | 1.00 (-) |
|  |  | Yes | 15 / 499 | 0.89 (0.50, 1.58) | 0.85 (0.47, 1.52) |

Abbreviations: CI, confidence interval; CLS-B, crown-like structures in the breast; HR, hazards ratio

^1^ Any CLS-B defined as detection of ≥1 CLS-B with adipocyte encirclement ≥75% on the tissue section examined

^2^ Adjusted for age at diagnosis (years), body mass index (kg/m^2^), and smoking status (never smoker, past smoker, current smoker)

^3^ Any CLS-B defined as detection of ≥1 CLS-B with adipocyte encirclement ≥90% on the tissue section examined

**References**

1. Lash TL, Fox MP, Fink AK. Applying quantitative bias analysis to epidemiologic data: Springer Science & Business Media; 2011.

2. Greenlee H, Shi Z, Hibshoosh H, et al. Obesity-associated Breast Inflammation among Hispanic/Latina Breast Cancer Patients. Cancer Prev Res (Phila). 2019;12(1):21-30. doi:10.1158/1940-6207.CAPR-18-0207

3. Iyengar NM, Chen IC, Zhou XK, et al. Adiposity, Inflammation, and Breast Cancer Pathogenesis in Asian Women. Cancer Prev Res (Phila). 2018;11(4):227-36. doi:10.1158/1940-6207.CAPR-17-0283

4. Iyengar NM, Morris PG, Zhou XK, et al. Menopause is a determinant of breast adipose inflammation. Cancer Prev Res (Phila). 2015;8(5):349-58. doi:10.1158/1940-6207.CAPR-14-0243

5. Iyengar NM, Zhou XK, Gucalp A, et al. Systemic Correlates of White Adipose Tissue Inflammation in Early-Stage Breast Cancer. Clin Cancer Res. 2016;22(9):2283-9. doi:10.1158/1078-0432.CCR-15-2239

6. Mullooly M, Yang HP, Falk RT, et al. Relationship between crown-like structures and sex-steroid hormones in breast adipose tissue and serum among postmenopausal breast cancer patients. Breast Cancer Res. 2017;19(1):8. doi:10.1186/s13058-016-0791-4

7. Vaysse C, Lomo J, Garred O, et al. Inflammation of mammary adipose tissue occurs in overweight and obese patients exhibiting early-stage breast cancer. NPJ Breast Cancer. 2017;3:19. doi:10.1038/s41523-017-0015-9

8. Fox MP, Lash TL, Greenland S. A method to automate probabilistic sensitivity analyses of misclassified binary variables. Int J Epidemiol. 2005;34(6):1370-6. doi:10.1093/ije/dyi184

9. Morris PG, Hudis CA, Giri D, et al. Inflammation and increased aromatase expression occur in the breast tissue of obese women with breast cancer. Cancer Prev Res (Phila). 2011;4(7):1021-9. doi:10.1158/1940-6207.CAPR-11-0110
